# Adjunctive use of hyaluronic acid in the treatment of gingival recessions: a systematic review and meta-analysis

**DOI:** 10.1007/s00784-024-05701-7

**Published:** 2024-05-21

**Authors:** Eleni Kalimeri, Andrea Roccuzzo, Alexandra Stähli, Ilias Oikonomou, Aaron Berchtold, Anton Sculean, Dimitrios Kloukos

**Affiliations:** 1 Department of Orthodontics and Dentofacial Orthopedics, 251 Hellenic Air Force & VA General Hospital, Athens, Greece; 2 Department of Periodontology, School of Dental Medicine, University of Bern, Bern, Switzerland; 3 Department of Periodontology, 251 Hellenic Air Force & VA General Hospital, Athens, Greece; 4 School of Dental Medicine, University of Bern, Bern, Switzerland; 5 Department of Orthodontics and Dentofacial Orthopedics, School of Dental Medicine, University of Bern, Freiburgstrasse 7, CH-3010 Bern, Switzerland

**Keywords:** Hyaluronic acid, Gingival recession, Root coverage

## Abstract

**Objectives:**

To explore the efficacy of Hyaluronic acid as an adjunctive in treatment of gingival recessions (GR).

**Materials and methods:**

A systematic literature search was performed in several electronic databases, including Medline/ PubMed, Embase, CENTRAL and LILACS. Recession improvement was evaluated through multiple outcome variables. The Cochrane Risk of Bias tool and the ROBINS-I tool were used to assess the quality of the included trials. Weighted Mean Differences (WMDs) and 95% confidence intervals (CIs) between test and control sites were estimated through meta-analysis using a random-effect model for the amount of Relative Root Coverage (RRC).

**Results:**

A total of 3 randomised studies were deemed as eligible for inclusion. Their data were also used for pooling the effect estimates. Overall analysis of RRC (3 studies) presented a WMD of 7.49% (*p* = 0.42; 95% CIs -10.88, 25.86) in favour of adjunctive use of hyaluronic acid during Coronally Advanced Flap (CAF) technique, although statistical significance was not reached. Statistical heterogeneity was found to be high (I^2^ = 80%).

**Conclusions:**

Within their limitations, the present data indicate that the local application of Hyaluronic acid does not lead to additional clinical benefits when used as an adjunctive to the treatment of GR with CAF. However, due to the high heterogeneity among the studies, additional well-designed RCTs are needed to provide further evidence on this clinical indication for the use of Hyaluronic acid.

**Clinical relevance:**

In the frame of the current review, the adjunctive use of Hyaluronic acid does not additionally improve the clinical outcomes obtained during treatment of GR with CAF.

**Supplementary Information:**

The online version contains supplementary material available at 10.1007/s00784-024-05701-7.

## Introduction

Gingival recession (GR) may become a major source of concern for both the practitioner and the patient; defined as an apical shift of the gingival margin with respect to the cemento-enamel junction, it leads to exposure of a root surface’s portion [1, 2]. This situation can be either localized or generalized and in conjunction to one or more tooth surfaces [3] and it has been related to several triggering factors such as traumatic toothbrushing, periodontal disease and orthodontic tooth movement [4–6].

As recession develops, several problems may also arise including compromised aesthetics, root caries and dental hypersensitivity [7], forcing patients to seek treatment.

Epidemiological studies have revealed that gingival recessions affect the majority of the adult population and in fact its prevalence, extent and severity seem to increase with age [8], thus making the management of recessions a serious concern for every clinician.

Several surgical techniques have been already proposed in order to address this clinical problem, by achieving complete root coverage, including coronally advanced flaps (CAF), laterally repositioned flaps and tunnel techniques [9, 10]. The existing evidence supports the concurrent use of CAF with sub-epithelial connective tissue grafts (SCTG) as the “golden standard” procedure for achieving optimal results [11]. Additionally, several biomaterials have been examined as possible adjunctive of root coverage procedures in order to avoid patient morbidity, such as enamel matrix derivative, collagen matrices and acellular dermal matrices [12–14].

Hyaluronic acid (HA), an anionic, non-sulfated glycosaminoglycan and a major component of the extracellular matrix in most tissues, is being used in various regenerative medical and tissue engineering approaches [15]. Recently, it has been studied as a possible adjacent treatment in periodontal [16] and implant surgery [17].

Several studies have shown HA’s positive effect on wound healing and tissue regeneration, by its properties of stimulating cell adhesion, migration and proliferation, mediation of cell signalling [18], clot formation, inducing angiogenesis, limiting bacterial contamination and stabilizing granulation tissue [19–22].

To the best of our knowledge, there is currently a lack of studies systematically evaluating all possible study designs that assess the possible benefits from the use of HA in root coverage procedures in order to provide the clinicians with up to date clinical evidence [23].

Therefore, the aim of the current systematic review was to explore the efficacy of hyaluronic acid as an adjunctive in treatment of gingival recessions.

## Materials and methods

### Registration of the study protocol

The study protocol was submitted to the PROSPERO international prospective register of systematic reviews hosted by the National Institute for Health Research (NIHR), University of York, UK, Center for Reviews and Dissemination and was allocated the identification number CRD42022321748.

### Reporting format

The latest (2021) Preferred Reporting Items for Systematic Reviews and Meta-Analyses (PRISMA) were adopted throughout the process of the present systematic review [24].

### Focused question and PICOS schema—Population (P), Intervention (I), Comparison (C), Outcomes (O) and study design

#### Focused question

In the surgical treatment of patients with gingival recessions, how efficacious is the adjunctive use of HA in comparison to standard treatment without supplementary use of HA, in terms of GR reduction?

#### Eligibility criteria

Studies meeting the following in inclusion criteria were included:

- **Population:** Patients with any type of gingival recessions in mandibular or maxillary teeth
- **Intervention:** Root coverage procedure with the adjunctive use of HA
- **Comparison:** Root coverage procedures without adjunctive use of HA
- **Outcomes:** GR reduction/ Complete Root Coverage (CRC)/ Relative Root Coverage (RRC)/ Recession Depth (RD)/ Recession Reduction (RR)/ Recession Width (RW) as primary outcome variables and gain in Clinical attachment level (CAL) / Periodontal pocket depth (residual or closure) (PPD)/ gain in Keratinized Tissue Width (KTW)/ Patient morbidity/ Change in bleeding on probing (BoP)/ Plaque Index (PI)/ Patient-related outcome measures (PROMs) such as pain, satisfaction, discomfort/ Quality of Life indicators and economic factors/ Root coverage Esthetic Score (RES), as secondary variables.
- **Study design:** Any study design was considered eligible for inclusion in this review, including randomized clinical trials (RCTs), non-randomized studies, prospective and retrospective studies. Case reports and case series were excluded. Follow up: All observation periods were accepted.

#### Exclusion criteria

i. Studies with insufficient information about the study design/ Pre-clinical studies/ Abstracts/ Letters to editors
ii. Studies that included individuals with systemic diseases.

#### Search strategy

Detailed search strategies were developed and appropriately revised for each database, considering the differences in controlled vocabulary and syntax rules by the last author (DK). No language or publication date restrictions were applied.

#### Electronic search

We searched the following electronic databases to find reports of relevant published studies up to 01.06.2023:

- The Cochrane Central Register of Controlled Trials (CENTRAL);
- MEDLINE (PubMed and via OVID);
- Ovid EMBASE
- LILACS

The full search strategy of Medline/ Pubmed is shown in Appendix 1.

#### Unpublished literature search

In order to further identify potential articles for inclusion, grey literature and possible ongoing trials were researched in the register of clinical studies hosted by the US National Institutes of Health (www.clinicaltrials.gov), the multidisciplinary European database (www.opengrey.eu), the National Research Register, and Pro-Quest Dissertation Abstracts and Thesis databases (https://about.proquest.com).

#### Manual search

Researchers engaging with the field were contacted in pursuit of additional relevant literature. All identified eligible studies ’reference lists were screened and manual searching of other published systematic reviews was conducted in order to obtain additional studies.

#### Study selection

All study selection steps were performed independently and in duplicate by two authors of the review (EK, EO), who were aware of study author identity, institution and study outcomes. Study selection comprised title-, abstract- and full-text-reading phases. After exclusion of non-eligible studies, discrepancies concerning the eligible studies were resolved by discussion with the third author of the review (AS). A record of all decisions on study identification was kept.

#### Data collection

Three authors were involved in data extraction. Data were initially extracted independently and in duplicate by the first two authors (EK, AB). Disagreements were resolved by discussion with the last author (DK). Data relating to the following study characteristics were collected: Author/ title/ year of study, study design, study aim, exclusion criteria, number/age/gender of patients, types of intervention across groups, follow-up period, outcome assessed, method of outcome assessment, measure of outcome, results and conclusion.

If stated, the sources of funding, trial registration, and publishing of the trial's protocol was recorded. This information was used to facilitate the assessment of heterogeneity and the external validity of the included studies.

#### Quality assessment

The methodological quality assessment of the included studies implemented the Revised Cochrane risk-of-bias tool (Rob 2) for the randomized trials [25] and the ROBINS-I tool for the non-randomised studies [26]. The studies were assessed individually and in duplicate by two reviewers (AR, AS) and their findings were compared. The last author (DK) was consulted to resolve any concerns on the quality assessment process.

#### Data analysis

Meta-analyses were conducted for studies reporting on similar interventions, comparisons and the same outcome measures in homogeneous populations. For continuous variables, mean differences and standard deviations were used to summarize the data gathered from each study. Regarding meta-analysis for continuous data, weighted mean differences (WMDs) and 95% Cls were calculated.

#### Heterogeneity

Examination of the study characteristics, the similarity between types of participants, the interventions, and the outcomes as specified in the inclusion criteria for considering studies for this review was performed in order to assess clinical and methodological heterogeneity. Statistical heterogeneity was reported by means of a Chi^2^ test and I^2^ statistic.

#### Assessment of reporting bias

Reporting bias arises when the nature or direction of the findings affects the reporting of research findings [27]. Potential reporting biases including publication bias, multiple (duplicate reports) publications and language bias were reduced by conducting an accurate and at the same time a sensitive search of multiple sources with no language restriction. Also, a search for ongoing trials was performed.

#### Subgroup analyses / Sensitivity analysis

As no sufficient data existed, subgroup analyses based on study characteristics or sensitivity analysis based on the risk of bias were not conducted.

#### Unit of analysis issues

We anticipated that some of the included studies presented data from repeated observations on participants, which could lead to unit-of-analysis errors. In such cases, we followed the advice provided in section 9.3.4 of the Cochrane Handbook for Systematic Reviews of Interventions [27].

## Results

### Description of studies

**Fig. 1:**
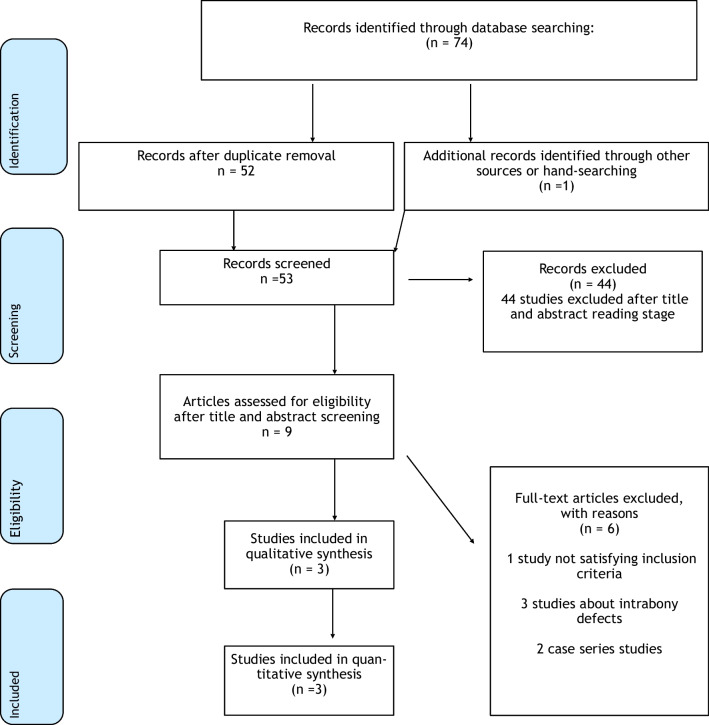
PRISMA flow diagram for study inclusion

The initial electronic search yielded 74 records. After title and abstract screening, 8 studies were further examined for eligibility in their full-text forms. In addition, 1 more study was identified through hand searching and was included in the study. After full-text reading, 6 studies were excluded, resulting in a total of 3 studies eligible for inclusion [28–30]. All 3 studies were RCTs (Tables 1 and 2). Two case series studies were excluded, although reporting relevant data, due to their design [31, 32]. The study selection process is presented in Fig. 1.

**Table 1:** Characteristics of included studies

Nr	Author (Year)	Study design	Follow up	Inclusion criteria	Exclusion criteria	Recession lesions	Test group or Group A		Control group or Group B	
							Intervention	Participants	Intervention	Participants
1	Pilloni et al. [28]	RCT, parallel design, single center	18 mos		Absence of a clearly identifiable CEJ, teeth with prosthetic crown or restoration with the cervical edge in CEJ area, smokers, active periodontal disease at sites, FMPS and FMBS ≥15%	At least one buccal Miller Class I/RT 1 recession (depth ≥ 2 mm) in the anterior area with at least 1 mm of keratinized tissue	CAF and HA Cross-linked HA (Hyadent BG) at tooth surface before flap suture	N: 15 Gender: 8 M, 7 F Median age: 30 y	CAF alone	N: 15 Gender: 8 M, 7 F Median age: 30 y
2	Rajan et al. [30]	RCT, split-mouth design	9 mos	At least 2 Miller’s class I or class II recessions at anterior teeth and first premolars, sufficient interdental bone radiographically, absence of pulling frenum in the keratinized tissue, no history of periodontal or mucogingival surgery in the past 6 months	Patients with systemic disease, history of smoking or drug allergy, smokers, patients with root surface restorations	Two or more sites of Miller's Class I or II recession on anterior teeth and first premolars	CAF and HA HA gel on root surface before flap advancement	N: 20 patients (20 sites) Gender: 7 M, 13 F Age range: 26–42 y	SCTG and CAF	N: same 20 patients (20 different sites) Gender: 7 M, 13 F Age range: 26–42 y
3	Kumar et al. [29]	RCT, split-mouth design, single-center	24 wks		Smokers, pregnant women, medically compromised patients, malaligned teeth, teeth in trauma from occlusion, teeth with cervical abrasions and restorations	Two or more sites of Miller's Class I recession on the canine and premolar region	CAF and HA Hyaluran gel (gengigel 0,2%) on root surface before flap advancement	N: 10 patients (10 sites) Gender: 7 M, 3 F Age: Not reported	CAF alone	N: same 10 patients (10 different sites) Gender: 7 M, 3 F Age: Not reported

*Mos* Months; *FMPS* Full-mouth plaque score; *FMBS* Full-mouth bleeding score; *Cl* Class; *MCAT* Modified coronally advanced tunnel; *HA* Hyaluronic acid; *SCTG* Subepithelial connective tissue graft; *LCT* Laterally closed tunnel; *N* Number; *M* Males; *F* Females; *y* Years; *RT1* Recession type 1; *RT2* Recession type 2; *RCT* Randomized clinical trial; *CEJ* Cementoenamel junction; *CAF* Coronally advanced flap; *wks* Weeks

**Table 2:** Outcomes of included studies

Author (Year) Study design	Outcome assessed	Method of outcome assessment	Measure of outcome	Results Mean value (± Standard deviation) [95% CI]	Conclusions
Pilloni et al. [28] RCT, parallel design, single center	1. RD 2. RR 3. PPD 4. CAL 5. MRC 6. CRC 7. KTW 8. Post-operative patient morbidity (7 days)	1,2. Periodontal examination, photographic records 2. Periodontal examination 3–5. Periodontal examination, photographic records 6. Number of teeth with CRC 7. Periodontal examination 8. Visual analogue scale questionnaire	1. Mean RD (mm) 2. Mean RR (mm) 3. Mean PPD (mm) 4. Mean CAL (mm) 5. MRC (%) 6. Incidence of teeth with CRC (%) 7. Mean KTW (mm) 8. Mean score (in swelling, discomfort and pain)	1. Significant decrease in RD in both groups, lower RD in test group (*p* < .05) 2. More reduction in test group [2,7 (± 1)] than in control group [1,9 (± 1)] (*p* < .05) 3. Not statistically significant differences (*p* = .717) 4. More improvement in CAL in test group [3 (± 1)] than in control group [2(± 1)] (*p* < .05) 5. 93,8 (± 13.08) % in test group, 73,1 (± 20.8) % in control group (*p* < .005) 6. 80% in test group, 33,3% in control group (*p* < .05) 7. Not statistically significant differences between groups (*p* = .116) 8. Less swelling and discomfort in test group (both with *p* < .05). Pain intensity not statistically significant (*p* = .151)	• HA effective in obtaining CRC for single Miller class I/RT1 gingival recession sites
Rajan et al. [30] RCT, split-mouth design	1. RD 2. RW 3. KTW 4. PPD 5. CAL 6. PI 7. GI 8. MRC	1–8. Periodontal examination	1. Mean RD (mm) 2. Mean RW (mm) 3. Mean KTW (mm) 4. Mean PPD (mm) 5. Mean CAL (mm) 6. Mean PI (%) 7. Mean GI (%) 8. MRC (%)	1. At 1 month, more reduction in test group [2.05 (± 0.69)] than in control group [2.45 (± 1.05)] (*p* < .05) with no other statistically significant differences between groups 2. No statistically significant differences between groups 3. No statistically significant differences between groups 4. At 3 mos, lower PPD in control group [1.10 (± 0.31)] than in test group [1.60 (± 0.68)] (*p* < 0.005) At 9 mos, lower PPD in control group [0.50 (± 0.51)] than in test group [1.15 (± 0.75)] (*p* < 0.005) 5. At 3 mos, higher level in test group [2.55 (± 1.10)] than in control group [3.05 (± 0.83)] (*p* < .05) At 9 mos, higher level in test group [1.90 (± 1.07)] than in control group [1.10 (± 0.91)] (*p* < 0.005) 6. No statistically significant differences between groups 7. No statistically significant differences between groups 8. At 3 mos, higher percentage in test group [58.43 (± 8.80)] than in control group [48.07 (± 13.35)] (*p* = .005) with no other statistically significant differences between groups	• Minor clinical differences between groups
Kumar et al. [29] RCT, parallel design, single center	1. RD 2. PPD 3. CAL 4. MRC%	Periodontal examination	1. Mean RD (mm) 2. Mean PPD (mm) 3. Mean CAL (mm) 4. MRC (%)	1. Not statistically significant difference (*p* = 0.00) 2. Not statistically significant differences between groups (*p* = .917) 3. Not statistically significant differences between groups (*p* = .2) 4. Higher in test group [68.33 (± 28,81)] than in control group [61,67 (± 30.22)]	• Not statistically significant differences between groups

*CRC* Complete root coverage (i.e., 100% root coverage); *PD* Probing depth; *CAL* Clinical attachment level; *MRC* Mean root coverage; *KTW* Keratinized tissue width; *RES* Root coverage esthetic score; *RD* Recession depth; *RR* Recession reduction; *mos* Months; *RW* Recession width; *PI* Plaque index; *GI* Gingival index

### Quality assessment

#### Randomized clinical trials

The RCT of Pilloni et al. was rated at an overall low risk, due to the low risk of bias that was applied to each domain based on the Cochrane risk of bias Robins 2 tool [28]. The RCTs conducted by Kumar et al. [29] and Rajan et al. [30] were judged to be at an overall high risk of bias, as the method of patient selection and allocation concealment were not described. Detailed assessment of the RCTs is provided in Table 3.

**Table 3:** Quality assessment of Randomized Controlled Trials

	Author/ Year	Study title	Bias arising from the randomization process	Bias due to deviation from the intended interventions	Bias in measurement of the outcome	Bias due to missing outcome data	Bias in selection of the reporting result	Overall Bias
1	Pilloni et al. [28]	Effectiveness of adjunctive hyaluronic acid application in coronally advanced flap in Miller class I single gingival recession sites: a randomized controlled clinical trial	Authors’ judgement: **Low Risk** Support for judgement: Use of a computer generated randomization list and sequentially numbered, opaque, sealed envelopes	Authors’ judgement: **Low Risk** Support for judgement: Carers and trial personnel aware of participants’ assigned intervention, but there were no deviations from the intended intervention beyond what would be expected in usual practice	Authors’ judgement: **Low Risk** Support for judgement: Outcome assessors blinded	Authors’ judgement: **Low Risk** Support for judgement: All outcome data available	Authors’ judgement: **Low Risk** Support for judgement: Reported outcome data unlikely to have been selected	Authors’ judgement: **Low Risk**
2	Rajan et al. [30]	Hyaluronon As An Adjunct To Coronally Advanced Flap For The Treatment Of Gingival Recession Defects	Authors’ judgement: **High Risk** Support for judgement: Sequence generation and allocation concealment process not described	Authors’ judgement: **Some concerns** Support for judgement: Carers aware of participants’ assigned intervention and there is no information on whether there were deviations from intended intervention	Authors’ judgement: **Some concerns** Support for judgement: No information provided about the blinding of outcome assessors	Authors’ judgement: **Low Risk** Support for judgement: All outcome data available	Authors’ judgement: **Low Risk** Support for judgement: Reported outcome data unlikely to have been selected	Authors’ judgement: **High Risk**
3	Kumar et al. [29]	Efficacy of hyaluronic acid (hyaluronan) in root coverage procedures as an adjunct to coronally advanced flap in Millers Class I recession: A clinical study	Authors’ judgement: **High Risk** Support for judgement: Quasi-randomized procedure with coin toss and patient selection not described. No details provided about allocation concealment process	Authors’ judgement: **Some concerns** Support for judgement: Carers and trial personnel aware of participants’ assigned intervention	Authors’ judgement: **Some concerns** Support for judgement: No information provided about the blinding of outcome assessors	Authors’ judgement: **High Risk** Support for judgement: Not all outcome data are available	Authors’ judgement: **High Risk** Support for judgement: The result being assessed is likely to have been selected, on the basis of the results, from multiple eligible outcome measurements (e.g. scales, definitions, time points) within the outcome domain	Authors’ judgement: **High Risk**

Bold: authors' judgment

#### Comparison of Complete Root Coverage (CRC)

The incidence of CRC was assessed in one study, where it was evaluated as the number of teeth with complete coverage of the recession defect after the root coverage procedure. The findings of Pilloni et al. in a RCT comparing the effect of application of HA in conjunction with coronally advanced flap (CAF) (test group, *n* = 15) to CAF alone (control group, *n* = 15) on single Miller class I/recession type 1 (RT1) gingival recessions, yielded that the application of HA provided increased probability of CRC. CRC was achieved in 80% of the test group and 33.3% of the control group (*p* < 0.05) [28].

#### Comparison of Relative Root Coverage (RRC)

The percentage of RRC, considering the reduction in recession depths at different time intervals, was assessed in all studies. The RCT of Pilloni et al. [28] found statistically greater improvement in RRC (*p* < 0.005) in the test group, which was treated with CAF and cross-linked HA application before flap suture (93.8 ± 13.08%), compared to the control group, which was treated with CAF only (73.1 ± 20.8%). RRC percentage was also higher in the test group in the 3-month clinical evaluation [test group: 58.43 (± 8.80)], control group: [48.07 (± 13.35)] (*p* = 0.005), in the RCT of Rajan et al. [30]. The test group was treated with CAF and HA, while, in the control group, a SCTG was harvested and CAF technique was performed. Likewise, a split-mouth RCT reported that higher RRC was obtained with CAF and HA application before flap advancement (68.33 ± 28.81 mm) than with CAF alone (61.67 ± 30.22 mm) in a 6-month follow-up examination [29].

#### Periodontal Probing Depth (PPD)

PPD was recorded in all the included studies. Even though Rajan et al. recorded lower probing depths in the control group during the 3- and 9- month examination, no other study revealed statistically significant differences regarding this parameter between groups at any of the time points [28–30]. It has been to be noted, though, that baseline PPD scores were significantly different between groups in this study.

#### Recession Depth (RD) and Recession Reduction (RR)

The depth of the recession defect, measured from the cemento-enamel junction to the gingival margin, was recorded in 3 RCTs. In the RCT of Pilloni et al., depth decreased more (*p* < 0.05) in the test group (CAF and HA application before flap suture) than in the control group (CAF alone), however, the median depth decreased significantly for both groups from baseline to 18 months after intervention [28]. According to the study of Rajan et al., more improvement in depth was observed in the test group (CAF + HA) in 1 month after intervention. No other differences were revealed between groups during the rest observation period [30]. Kumar et al. in their split-mouth RCT reported that the differences in recession depth values between groups were not statistically significant [29].

RR was evaluated as a separate clinical parameter in the study of Pilloni et al., where more reduction was attained in the test group (CAF and HA application before flap suture) (2.7 ± 1 mm) than in the control group (CAF alone) (1,9 ± 1 mm) from baseline to the 18-month evaluation [28].

#### Clinical Attachment Level (CAL)

Measurements of CAL were obtained in three studies at baseline and follow-up examinations and were calculated as the sum of PPD and RD at the midfacial site of the tooth. Pilloni et al. demonstrated a greater improvement in CAL (*p* < 0.05) in the group treated with CAF and HA application (3 ± 1 mm) than in the control group (2 ± 1 mm) [28]. Similarly, in the study of Rajan et al., CAL measurements improved more for the test group (CAF + HA), when compared to those of the control group (SCTG + CAF) [3 months: higher level in test group [2.55 (± 1.10)] than in control group [3.05 (± 0.83)] (*p* < 0.05) and 9 months: higher level in test group [1.90 (± 1.07)] than in control group [1.10 (± 0.91)] (*p* < 0.005) [30]. In the contrary, Kumar et al. found no significant differences in CAL between the test (CAF and HA) and control group (CAF alone) at follow-up examinations [29].

#### Keratinized Tissue Width (KTW)

KTW was investigated in two studies and was calculated as the distance between the gingival margin and the mucogingival junction at the midfacial point of each tooth. In the RCT conducted by Pilloni et al., no differences were found concerning the KTW between baseline and follow-ups or between test (CAF and HA) and control (CAF alone) group [28]. The differences between the test (CAF + HA) and control group (SCTG + CAF) were also non-significant in the study of Rajan et al. [30].

### Post-operative patient morbidity

Patient morbidity was evaluated by Pilloni et al. through Visual Analogue Scale questionnaires at 7 days after intervention. With respect to pain intensity, there were no differences between the test (CAF and HA) and control group (CAF alone), while swelling and discomfort were rated as lower in the test group (*p* = 0.010 and *p* = 0.029, respectively) [28].

### Quantitative synthesis of included studies

**Fig. 2:**
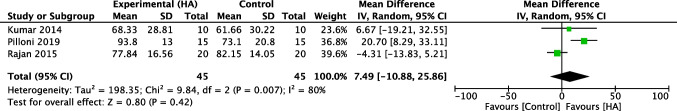
Forest plot of HA in recession treatment (Outcome:RRC in 6 months)

A meta-analysis was only feasible for RRC. Despite the methodological heterogeneity, mainly in terms of follow-up, we have decided to report the meta-analysis and highlight its limitations. Data from 3 studies were used for meta-analysis [28–30]. Overall analysis of RRC presented a WMD of 7.49% (*p* = 0.42; 95% CIs -10.88, 25.86) in favor of adjunctive use of hyaluronic acid, although statistical significance was not reached. Statistical heterogeneity was found to be high (I^2^ = 80%) (Fig. 2).

## Discussion

The aim of the present study was to assess the level of evidence on the use of HA in the treatment of gingival recessions. Based on the best available evidence (i.e. three RCTs) no conclusions could be drawn with respect to the adjunctive clinical efficacy of HA.

It is well documented that to provide the best outcomes in terms of mean and complete root coverage, as well as to increase keratinized tissue, SCTG is the most effective therapeutic approach for Miller Class I and II single-tooth recessions, as concluded in the consensus report of the 2015 AAP Regeneration Workshop. Nevertheless, following the harvesting procedure, some post-operative sequelae such as patient’s discomfort have been reported [33]. Consequently, the use of alternative biomaterials, such as acellular dermal matrix graft [34] or enamel matrix derivative [35] have been proposed to serve as alternatives to autogenous tissue and have been described as less painful, whereas use of palatal tissue as a donor site has been related to increased complications [33]. More specifically, research concerning gingival recession treatment always focused on exploring convenient alternatives to reduce patient morbidity and maximize the intervention predictability [36–39].

A critical factor determining the outcome of root coverage procedures, apart from excellent surgical technique, is the undisturbed wound healing process. Recently, the application of HA on the surgical area has been proposed based on its characteristics which promote the early wound healing phases; during the inflammatory phase of wound healing, HA has been shown to enhance inflammatory cell migration, proinflammatory cytokine production and stabilization of granulation matrix. Thereby, HA exerts its effects through two main routes, firstly via HA receptors such as CD44 that are present on cell membranes of nearly all human cells and by providing a wound-microenvironment that enables optimal healing [40, 41]. Subsequently, in the granulation phase, HA promotes the extracellular matrix cell proliferation and migration, and facilitates angiogenesis. Lastly, HA aids in epithelium formation [42]. Thus, it is expected that the addition of HA to root coverage procedures will have a positive effect in terms of CRC and RRC increase.

The ideal goal of every mucogingival surgical intervention is to obtain the CRC [43, 44]. HA application combined with a CAF, CRC was detected in a higher percentage of cases compared to CAF alone [28]. On the other hand, other studies which applied HA in combination with a SCTG and a tunnel technique, reported a CRC in 50 and 20% of single and multiple recessions [31, 32]. A plausible explanation for this difference is the fact that treating multiple recessions is often a more demanding surgical procedure with increased risk of complications during the healing process [45].

When focusing on the RRC, 2 out of the 3 RCTs reported better results in the test compared to the control group, supporting the assumption that HA may facilitate a more favorable wound healing process and, thus, more satisfactory clinical results (Fig. 2). Considering the study of Rajan et al. [30], that was included in this meta-analysis, it has to be noted that the patient oral hygiene motivation may decrease significantly after 6 months post-op; in that frame, late success may not always be achievable.

When focusing on the KTW, no differences were detected in the studies of Pilloni et al. [28] and Rajan et al. [30]. With respect to the periodontal parameters (i.e. CAL and PD), in the RCTs of Pilloni et al. and Rajan et al., more improvement was observed in the test groups [28, 30]. The explanation of this difference may again lie on the basis of the HA biological properties on wound healing modulation [16, 42, 46]. On the other hand, Kumar et al. reported no differences in CAL between the groups (CAF and HA before flap advancement, CAF alone) at the 24-week follow-up, even though the CAL gain was significant for both groups [30]. In contrast to some studies which reported positive results in terms of PPD reduction following non-surgical periodontal treatment and local application of HA [47, 48], the present studies did not detect statistically significant changes at any follow-ups.

One of the aspects that has to be taken into consideration when performing mucogingival procedures, is patient morbidity and mainly pain perception [49]: in the study by Pilloni and co-workers [28], the application of HA resulted in statistically significant, although minor, improvements in swelling and discomfort, as compared to the control group. These outcomes may again be explained by the angiogenic properties of HA, its role as a hydrating active ingredient and its contribution in the regulation of inflammation, through enhancing the lymphocyte, inflammatory, and connective tissue cell motility [20, 42, 46, 50–52].

Nevertheless, it has to be pointed out that despite the biologically plausible effects of HA with respect to wound healing and regeneration, the clinical evidence for the adjunctive application of HA to recession coverage surgery is still very limited. A recent systematic review, including 3 studies, also reached the same conclusion [53].

## Limitations

Limitations of this review were foremost the shortage of studies eligible for inclusion, as well as the included studies’ design. The meta-analysis included RCTs with various follow up periods: 6 months for the study of Kumar et al. [29], 9 months for the study of Rajan et al. [30] and 18 months for the study of Pilloni et al. [28]. Despite this fact, the review team has accepted 6 month follow up as adequate to retrieve clinically significant results.

## Conclusions

Given the limited available evidence, as well as the paucity of high-quality evidence, valid inferences concerning possible effect of HA on root coverage procedure could not be drawn. Therefore, further research is required to improve the quality of evidence concerning this topic.

### Supplementary Information

Below is the link to the electronic supplementary material.

Supplementary file1 (PDF 21 kb)

## Data Availability

The data that support the findings of this study are available from the corresponding author upon reasonable request.
