# Supplementary material for: Adjunctive use of hyaluronic acid in the treatment of gingival recessions: a systematic review and meta-analysis
Source: Clin Oral Investig. 2024 May 21;28(6):329. doi: 10.1007/s00784-024-05701-7 (PMC11108902; doi:10.1007/s00784-024-05701-7)
Supplement: Supplementary file 1 — Supplementary file1 (PDF 21 kb) [file 784_2024_5701_MOESM1_ESM.pdf]

PubMed Search History- 01.06.2023

| Search number | Search Details |
|---|---|
| 1 | "hyaluronic acid"[MeSH Terms] OR ("hyaluronic"[All Fields] AND "acid"[All Fields]) OR "hyaluronic acid"[All Fields] |
| 2 | ("hyaluronic acid"[MeSH Terms] OR ("hyaluronic"[All Fields] AND "acid"[All Fields]) OR "hyaluronic acid"[All Fields]) AND ("gingival recession"[MeSH Terms] OR ("gingival"[All Fields] AND "recession"[All Fields]) OR "gingival recession"[All Fields]) |
| 3 | "gingival recession"[Title/Abstract] AND "hyaluronic acid"[Title/Abstract] |
| 4 | "gingival recession"[MeSH Terms] AND "hyaluronic acid"[MeSH Terms] |
| 5 | ("Hyaluran"[All Fields] OR ("hyaluronic acid"[MeSH Terms] OR ("hyaluronic"[All Fields] AND "acid"[All Fields]) OR "hyaluronic acid"[All Fields] OR "hyaluronan"[All Fields] OR "hyaluronans"[All Fields])) AND ("gingival recession"[MeSH Terms] OR ("gingival"[All Fields] AND "recession"[All Fields]) OR "gingival recession"[All Fields]) |
| 6 | "gingival recession"[MeSH Major Topic] AND ("hyaluronate"[All Fields] OR "hyaluronates"[All Fields] OR "hyaluronic"[All Fields]) |
